# Book Reviews

**Published:** 1811-02

**Authors:** 


### CRITICAL ANALYSIS
OF
### RECENT PUBLICATIONS
IN THE
DIFFERENT BRANCHES OF PHYSIC, SURGERY, AND MEDICAL PHILOSOPHY,

---

### *Transactions of the Medical Society of London.*

(Concluded from p. 63 of the Journal.)

MR. WARE, one of the Vice Presidents, has favoured the Society with an interesting account of some diseases of the eye, which forms the subject of the 6th article. The first of these he styles *staphyloma*, this term has been used to designate the protrusion of a part of the iris through a wound or ulcer of the cornea; but Mr. Ware uses the term to denote a projecting opaque cornea.

"It has been disputed by authors (he remarks, p. 116) whether the projection of the opaque cornea in the staphyloma, is occasioned by a thickening of this tunic, or by a morbid accumulation of aqueous humour behind it. I believe, in general, both these circumstances combine to produce the disorder; the cornea becoming not only opaque, but softer and thicker than in its natural texture, and, in consequence of this, the aqueous humour behind the cornea pushes it forward, and thus enlarges the anterior chamber of this humour. I have sometimes seen the whole cornea sloughed off during an acute purulent ophthalmy, and a white opaque substance gradually effused from the ulcerated surface, sufficient to form a complete cover to the iris; after which this opaque body has gradually projected in a conical shape, until at length it has become so prominent as to hinder the eyelids from closing over it. I have at other times seen the projecting cornea partly opaque, and partly transparent: the pupils being distinctly visible through the transparent part, but the power of vision wholly destroyed. Sometimes the circumference of the opaque cornea projects, its central part appearing depressed, and resembling the bottom of a plate or dish; and sometimes near to the center of opacity, in the last case mentioned, there is an irregular black appearance, which a cursory ob erver might mistake for a pupil. No part of this aperture, however, is perceptible on a careful inspection, and the eye of course is deprived of all useful vision.

Mr. Ware next considers the means of affording relief. When the projection of the opaque cornea can be covered by the eyelids, without painfully stretching them, if the surface of the cornea continue regular, and the sight of the other eye perfect, no inconvenience, except the unseemly appearance, is produced. This may be remedied by wearing spectacles containing plain window glass in the ring opposite the sound eye, and glass that is ground in a slight degree opaque, in the ring opposite the affected eye. In some cases it is necessary to sink the eye. For this purpose caustics and other strong applications have been proposed; but Mr. Ware deems them dangerous and inadequate. The mode of compressing the tumour, he states, is difficult, and he only remembers one case in which it afforded advantage.

"The more direct way of affording relief in the staphyloma is by removing the whole of the projecting substance; in consequence of which the humours of the eye are discharged, and the posterior part of its tunics collapse, so as to form a kind of button at the bottom of the orbit. On this button, when the wound is healed, an artificial enamelled eye is capable of resting; by which the uniform appearance of the face may be restored."

Our author proceeds to enquire into the best mode of performing the operation. He objects to the method of passing a double ligature through the tumour, and tying it on each side, as practised by Neister, St. Yves, and others, because it is a painful and indirect mode of accomplishing our object. He thinks Scarpa's method of removing a small portion only of the projecting cornea, and forcing out the crystalline and vitreous humours, is liable to considerable objections, for which, however, we must refer to his paper, and content ourselves with giving Mr. Ware's own mode of operating, which he informs us has uniformly succeeded in a considerable number of cases during a practice of more than thirty years.

"The operator is directed to stand behind the patient, who is to be placed on a chair, sufficiently low to allow the operator to carry his hand with ease over the patient's head. A large crooked needle, armed with a strong thread, should then be passed through the opaque projecting cornea, and after separating the needle from the thread, a knot should be tied in the latter, at a small distance from the eye, in order to hinder the thread from slipping. The operator having thus obtained, by means of the thread, a secure hold of the eye, a knife similar to that which is used to divide the cornea in extracting the cataract, or, if this be not at hand, a long sharp-pointed lancet, should be pushed through the sclerotic coat, about a quarter of an inch from its connection with the cornea, and be carried quickly but accurately round the cornea, as nearly parallel to it as can be accomplished. Sometimes, as soon as a puncture is made through the sclerotica, so large a portion of the vitreous humour escapes, as to cause the cornea to become flaccid, in consequence of which the operator may find it difficult to complete the incision sion round this tunic with either the lancet or the knife, and in this case a curved blunt-pointed scissars will be found useful to finish the operation. The only objection to the use of the scissars is drawn from the additional pain which it is supposed to give; but the duration of the operation is so short, that the difference between the pain produced by the instruments is scarcely worthy to be named. The hæmorrhage that succeeds is seldom considerable; and the less the eye is examined afterwards, the less danger will there be of pain and inflammation. A compress wet with a saturnine lotion should be applied over the eye, and it should be moistened as often as it becomes dry; but no lint or any other application should be put within the lids, since this has been known to give great pain, and in one instance to occasion alarming symptoms. An anodyne should be given after the operation, of greater or less strength according to the age of the patient; but it is seldom necessary to repeat this medicine, since the patient has usually more sound and quiet sleep after the operation than he had for a long time previous to its performance. At the end of about a fortnight, that part of the sclerotica which remained in the orbit will be found to have collapsed, and sometimes a small fungous substance will then protrude through the wound. This in the course of time would subside of itself, but, as the delay may be irksome, the fungus may be easily removed, and with very little pain, by nipping it off with a pair of sharp scissars. The fungus is usually smaller in its neck where it joins the sclerotica than in its top; in consequence of which its removal is effected with very little difficulty; and though it sometimes reappears, it may be nipped off again and again, until at length the wound will completely close, the inflammation cease, and the orbit become fit to receive an artificial eye. This, however, ought not to be introduced until the inflammation be perfectly removed, and when such an eye is used, it is adviseable to withdraw it every night and replace it in the morning, which may be effected with ease by the patient himself after a short experience. P. 127.

Hydropthalmia, to which Mr. Ware next calls our attention, is an enlargement of the whole eye, produced by an increase of the vitreous as well as of the aqueous humour, which causes the eye to occupy an undue portion of the orbit, and occasions pain when the eye-lids are closed over it. Children are sometimes born with the corneæ large, prominent, and opaque; these, in general, are removed as the child grows older, by what Mr. Ware terms, "the *vis naturæ medicatrix*," without any particular remedy being employed. But when the enlargement is not confined to the cornea, and extends to the sclerotica, and the eye-lids cannot be closed without difficulty; the patient being at the same time blind, and unable to sleep without opiates, it becomes necessary to devise some means of affording ease, and obviating deformity; for the prospect of restoring sight is lost. For this purpose we are recommended to perform the operation for diminishing the eye, as directed in cases of staphyloma.

"Before an operation of so much importance be performed, it is, however, essentially requisite to ascertain that the disease consists solely in

 an an enlargement of the different parts of the eye; and that it is not produced by the formation of purulent matter within the eye; by a morbid alteration in the structure of either its coats or humours; nor by the undue accumulation of adeps, or of any other substance, behind this organ." P. 134.

Some cases illustrative of Mr. Ware's mode of discriminating these affections, and his method of treatment are related. For his account of Carcinoma, we must refer to the volume itself, having already advanced too far beyond our limits.

The 7th article, by Mr. Burns, of Glasgow, is a case of suppuration of the liver, with appearances resembling Ascites, which terminated favourably. A girl aged twelve, on the 20th of June, 1798, complained of sickness, pain about the upper part of the abdomen, shooting to the right shoulder, heat, and frequency of pulse. An emetic was given; the pained parts were rubbed with some embrocation, and saline draughts were prescribed. The pain continuing, on the 10th of July, a blister was applied over the lower ribs, and was repeated on the 15th without much advantage. For some days previous to this, the belly had been fuller than usual; and the swelling having increased, diuretics were ordered. Mr. Burns saw her, for the first time, on the 19th of July, and says,

"I found the right hypochondrium tumid and painful to the touch, especially near the stomach, and toward the lower part. She had pain in the right shoulder, and could not lie with ease except on her back, or inclining to the right side. The tongue was furred, the tunica adnata of the eye was of a yellowish colour; the urine was high coloured, and deposited a copious pink coloured sediment. The belly was bound, and the stools, when procured, were of a light colour. The appetite was much impaired, and she had frequent fits of sickness and retching. The pulse was 115, sometimes 120; the body emaciated, and the skin wet with perspiration, whilst a hectic blush pervaded the cheek. The belly was considerably swollen, and a fluctuation could be discovered. The tumor had not so diffused an appearance as is observed in Ascites, but was more rounded, as if a large globe had been placed below the umbilicus.

"She was now put on a course of mercury which was continued three weeks, so as to keep the mouth somewhat sore. During this time the symptoms of hepatic inflammation went off, she slept better and felt easier; but the hectic symptoms continued, and, although the diuretics had not been discontinued, the belly increased in size."

On the 12th of September, she was tapped in the usual way, and about six pounds of well conditioned pus were drawn off. After this she soon got entirely well. In the middle of October the belly again swelled, but not to half its former size; the umbilicus protruded and inflamed: a poultice was applied, and in a few days the skin burst, and nearl nearly two pounds of matter were discharged. Pus continued to ooze out of the aperture for a fortnight, after which it healed up, and the patient has remained in good health ever since.

Observations on the hare-lip, by Isaac Rand, form the subject of the 8th article. This paper was read in 1797, since which two volumes of the Society's memoirs have appeared. Perhaps the editors of the former volumes were startled at the author's first paragraph, which seems to us somewhat paradoxical. He advances the following curious position, "Natural deficiencies are more frequent in the upper lip than in any other part of the body; but, happily, these are very rare." What are very rare? Deficiences in the upper lip, or in the other parts of the body? The object of the paper is to demonstrate the propriety of operating at a very early age in hare-lip. In two cases Mr. Rand performed the operation two days after birth. The first case was going on very finely, when an unlucky diarrhœa arrested "the efforts of nature, in completely uniting the bones in the middle and posterior parts of the fauces," and "destroyed the child in its seventh month." The other case, however, completely succeeded; as did another in which the operation was performed at three months. The advantages which result from performing the operation at an early period, the author observes, are many.

"Children, for some days after birth, seem to be in a torpid state, and in general require but little nourishment. They are easily managed, and make no opposition during the operation. If there is only a fissure in the lip, and sometimes when there is one in the bones, immediately after the union of the fissured part of the lip, the child is capable of sucking, by which the chance of surviving the infantile state is increased. The disagreeable impression upon the mother's mind, of propagating the deformity in the family by future births, is removed." P. 165.

At this advanced period of the surgical art, such a paper as this presents no novelty in practice, and we find nothing in it to induce us to commend the diligence of the present Editors, in disturbing its quiet repose in the Society's treasury of manuscripts.

The next article, by Mr. Norris, contains the histories of two extraordinary cases. The first is that of a lady, aged 51, who, about the middle of April, 1803, in going down the cellar stairs, fell and pitched with her forehead against a heap of wood. She was sick and faint for a short time, but on the following and succeeding days, thought little of it. In the beginning of June, she had an erysipelatous inflammation in the arm. In July she was affected with violent pain in the head, which continued, without alleviation, though Dr. Nankivel prescribed for her during more than a fortnight.

fortnight. August the 2d, she consulted Mr. Upton, who "found her labouring under a smart active fever; a hot and dry skin; quick pulse, with considerable anxiety and agitation; and complaining of violent pain in her head generally, but especially on the os frontis. Upon this bone was a tumour with a large hard base, terminating in a conical point, exactly resembling the growing horn of a young heifer. This evidently contained a fluid, which on the following day was set at liberty with the point of a lancet, and upon examination, a considerable surface of the bone was found bare." P. 170.

Mr. Norris saw this patient on the 9th of August. He enlarged the opening on the scalp, advised the head to be fomented, and the bowels to be kept open. At the end of a week, no abatement in her suffering having taken place, he applied the trephine. When the operation was over, he was surprized to find that the dura mater exhibited a perfectly healthy appearance; and that no matter was discharged than what oozed from the substance of the bone. Some alleviation of pain followed, which he attributed to the medicines that were prescribed.

In the course of a week, she complained of pain toward the posterior and upper part of the left parietal bone, and a little tumour, tender to the touch, and containing a fluid, was observed there. On opening it, the bone was found bare. "After a few days, this portion of bone, of the size of a sixpence, separated, leaving a healthy granulating surface, and the wound very soon healed."

From this time, similar tumours, with similar results, occurred almost daily. During the first two or three months the separated pieces of bone were from the outer table of the scull; after that time the dura mater was generally exposed by the separation of each piece, and the wounds in the scalp no longer healed. She died, worn out with suffering, on the 10th of May, 1804.

This patient had not one other, even doubtful, symptom of syphilis, and from Mr. Norris's inquiries, he thinks it certain that she was never affected with that disease. A plate accompanies the description, and the fragment of scull which remains has the appearance of being worm-eaten.

For the second case, de *Satyriasmo*, we must refer our readers, at least, those who understand Latin, to the volume itself. The tenth article is "on the Medicinal properties of Sanguinaria Canadensis, or Blood Root. Communicated to the President by Dr. N. Smith, Hanover (North America). Dated February, 1807. Its medical virtues are very considerable. "The dried root, pulverised and given in doses of four or five grains, generally pukes pretty violently, producing a great prostration of strength during its operation, which continues for some time." "The taste is acrid and unpleasant.

sant. The pulverised root taken into the nose excites sneezing, and produces a sense of heat in that organ. It also acts as an escharotic on fungous flesh." Dr. Smith has not known it act as a cathartic. He has cured several polypi of the soft kind, by the continued use of it as a snuff. He has given it with great success in hæmoptysis, and in coughs.

"From more than two years experience of the use of blood root in affections of the lungs, attended with cough, I cannot assert (the Doctor observes) what has been asserted of fox-glove, that it will cure a confirmed consumption; but I can in confidence say, that, in my opinion, it is capable of doing more towards preventing that fatal disease than any one remedy I have ever been acquainted with.

"I have given blood root in powder, in tincture, and in simple infusion; which last is the better mode of giving it. In powder it operates more roughly, and spirit does not appear to extract its active principle sufficiently. When I give it for a cough, if the symptoms are urgent, I begin with a dose sufficient to excite puking; but generally endeavour to give it in as large doses as can be borne without that effect, and repeat it four or five times each day. Where there is great irritation and a constant disposition to cough, I join opium with it. Given in this manner, if the patient has not a confirmed hectic, it generally cures the cough." P. 184.

It is also useful in inflammatory rheumatism. It has been recommended in jaundice, and has cured epilepsy. It has never been known to produce any lasting bad effects, and never affects the head like fox-glove. If then it be not a valuable addition to our Materia Medica, it may at least become the basis of a profitable nostrum. Mr. Mason Good has subjoined an accurate botanical description of the plant.

The next article is a short and very unsatisfactory case of Tic Douloureux, by Dr. Anthony Fothergill, of Philadelphia. Most of the medicines usually prescribed in this painful complaint were given without any benefit. The operation of dividing the nerve was proposed and rejected; and for any thing we know to the contrary, the patient is still afflicted with the disease.

The 12th article contains "*Remarks on the Land Winds and their Causes*," by William Roxburgh, M. D.

In the 13th are related "Cases illustrating the effects of oil of turpentine in expelling the tape-worm." By Drs. Lettsom, Hancock, Fothergill, Birkbeck, and Mr. Saner. The result of these cases establishes the efficacy, and confirms the safety, of a mode of practice which has only lately been known. Dr. Lettsom gave nine drams of the rectified oil of turpentine, desiring the patient to swallow a little honey after it. The medicine occasioned less heat than would have been occasioned by as much brandy or other spirit, and the flavour vour and heat were removed by the honey. "In about three hours after taking this dose, a laxative motion was produced, without any discharge of tænia: but soon afterwards, with the second stool, more than four yards of the worm were discharged, and also a quantity of matter, resembling, as the patient expressed it, the substance and skins of the tænia." No pain or uneasiness was experienced in the urinary passages after taking the medicine, and the patient has since remained in perfect health. Dr. Lettsom concludes that the best mode of taking the oil is without admixture. Dr. Hancock began by prescribing the oil of turpentine in doses of two drams twice a day, mixed with treacle. "This produced no other effect than an increase of pain and uneasiness, and particularly on going to stool, as if it irritated the rectum. The dose was now increased to half an ounce, at longer intervals. The first dose in this quantity, which she took without treacle, produced a little sickness and confusion of ideas, and afterwards operated as a purge. She complained of no uneasiness whatever in the urinary organs." After these doses, she passed a quantity of slimy mucus, and obtained so much relief in all her painful symptoms that she begged for a double dose. She accordingly took an ounce of the oil which produced slight intoxication, till the cathartic effect followed. This was repeated several times without, however, any appearance of tænia in her stools, though the dose was increased to an ounce and a half. Dr. Hancock observed that the mucus which was abundantly discharged by the operation of the medicine, "sometimes exhibited the appearance of white films, as if the substance of the worm had been broken down."

Dr. Fothergill gave a patient affected with tænia half an ounce of the oil of turpentine. It was taken in tea, sweetened with honey. "In a quarter of an hour he (the patient) was seized with retching, and in the course of the day passed four copious stools, in one of which was a tape worm several yards in length." It was dead and had a livid appearance. "The dose of the oil was increased to six drams, and was repeated twice a week for the space of a month. During the first fortnight small pieces of worm continued to pass away, both after taking the medicine and at other times; but in the second fortnight the stools were natural, and contained no vestige of tænia." The remedy was consequently discontinued, and the patient regained his strength and cheerfulness with an entire freedom from complaint.

Dr. Birkbeck administered the oil of turpentine to two middle aged females who had long been troubled with the tapeworm. In the first case, half an ounce was given, and produced two evacuations from the bowels, in one of which more more than four yards of the worm were contained. "It was dark-coloured, shrivelled, filmy, and lifeless." A second dose of the oil did not expel any more of the worm, nor had it again appeared three months afterwards. "Considerable derangement of the general health and great pain in the pit of the stomach were produced by the tape-worm, in the second case in which the oil of turpentine was employed. Although one tea-spoonful only was introduced, sickness and acute pain followed: this dose was repeated several successive mornings, always with the same immediate effects; but occasionally it was succeeded by the expulsion of large portions of the worm." After continuing the medicine some time, the patient became free from any appearance of tænia in the stools, and from all those sensations which had so long denoted its presence in the intestines.

Mr. Saner gave an ounce of the rectified oil of turpentine, with an equal quantity of syrup of saffron, to a woman who had long been troubled with tænia, portions of which for seven years past had come away, whenever she took a dose of jalap. Several feet of the worm, with the head attached, speedily came away, and she has remained well ever since. The second case was not so favourable. One dose of the oil of turpentine brought away a quantity of worm, without occasioning any unpleasant effects; but on repeating it, it produced "violent retchings, tenesmus, strangury, and great pain in the back; the urine was also a little tinged with blood." The patient afterwards took a drachm of jalap, and passed a considerable quantity of the worm. From the evidence of these histories, and a variety of other cases with which we are acquainted, we have no doubt that the large doses of oil of turpentine may be given with safety, and in general will succeed in expelling tænia from the bowels.

The volume concludes with an account of the life and writings of Dr. Hulme, by Dr. Clutterbuck.

---

*Additional Cases, with further Directions to the Faculty, relating to the use of the Humulus, or Hop, in Gout and Rheumatic Affections.* By A. Freake, Apothecary, 8vo. sewed, pp. 43. Highley.

Nearly six years have elapsed since Mr. Freake gave, in this Journal, an account of the medicinal qualities of the Hop. As his experience extended, in 1806 he was enabled to form a pamphlet, which contained some interesting facts on the subject. The Royal College of Physicians has subsequently introduced two preparations of the medicine into the last edition of their pharmacopœia, and we doubt not that they will prove efficacious in many cases. Mr. Freake has adduced some additional instances of the success of the remedy, one of which we quote as a specimen.

*April* 13, 1806. This day I was sent for to a gentleman 46 years of age, much troubled with Gout. I found him on a sofa complaining of violent pain in his left ancle, which was swelled but did not appear inflamed, tongue white, pulse 96, skin hot and dry, his nights were restless, and his spirits very low. He had a violent shock the preceding day from being thrown out of a chaise, by which accident he had a slight contusion in his right knee, yet he was able to use considerable exertion in the evening, and did not feel pain or uneasiness in the left ancle until this morning. He had not long recovered from a fit of Gout, which had confined him for some time, as it usually had done two or three times in each preceding year. I directed for him some aperient medicine, and then gave him the Humulus with saline draughts; these had the usual effect of throwing off more Gout, promoting perspiration, procuring rest, and keeping the bowels gently open, at the same time lessening the frequency of the pulse. The symptoms varied occasionally, Gout attacked different limbs, and afterwsrds subsided kindly; the patient now took the medicines regularly, without the saline, and amended so rapidly, that on the

19th, I was able to declare him in a convalescent state; the bowels were not now sufficiently active, therefore I ordered an aperient draught, which procured three motions, and on the

20th, When I called, my patient informed me he was better in every respect, that he was happy to say, I had done him a great deal of good. He had slept well, his tongue was clear, pulse 76, his appetite had increased, and his spirits were very much improved, the swellIng and inflammation had subsided, and he had got on his small gout shoes, and was able to use considerable exertion: the medicines were continued.

27th, Last night he was in such health and spirits, that he exerted himself with increased agility, and unfortunately strained the instep of his left leg, which occasioned considerable uneasiness and pain, and brought on fever, which, by the addition of saturated lemon juice to his Humulus medicine, he was relieved from on the following day.

28th, He is now considerably better, and has gone through a very fatiguing evening. The medicines were repeated.

30th, Ordered the draught and pills to be taken only at bed time.

*May* 3. This day, I received the thanks of my patient and his wife, on his being restored to better health than he had experienced for many months past.

7th, I called to see my patient, who remained well, but had not fortitude sufficient to persevere with the medicine when out of pain. He, however, was free from Gout for nearly *eighteen months*.

By persevering for some time in the use of the remedy, after the complaint is removed, its future accessions have been rendered less violent, till at length, the patient has become altogether altogether free from the disorder. Mr. Freake gives the following directions for administering the medicine.

### DIRECTIONS.

In acute Gout, after the bowels have been cleared, the patient cannot too soon begin the use of the Humulus. Ten grains of extract should be formed into pills, with rhubarb and ginger for a dose; a drachm of the tincture should be added to a saline draught to be taken after the pills, or the pills may be dissolved in the draught; this draught with the pills should be repeated every four hours, and double the quantity of extract and tincture may be put into the night draught if the symptoms are urgent.

The medicine thus administered, has in general given relief in one, two, or three nights. This plan must be continued every four or six hours, while fever is present; after which the saline may be lessened, as well as the number of doses of the Humulus for a few days. A decoction of the Peruvian or Cascarilla bark may then be substituted for the saline draught, and continued two or three times a day, until strength is regained, or for about a fortnight after the symptoms of Gout have subsided. In some cases, when I could not prevail on my patient to take the number of doses, I have made the medicines proportionably stronger, and given them less frequently, allowing a drachm of the extract, and six drachms of the tincture in each twenty-four hours, as the extent of quantity.

The regimen in acute Gout should be similar to that recommended in inflammatory fever.

Attention must be paid to keep the bowels gently open, if the medicine is not sufficient for that purpose.

The above plan has succeeded in acute Rheumatism, assisted by the lancet when needful, and occasionally repeating the aperient medicine.

In chronic Gout the medicines must be given in a very different manner, and for a much longer time, for two or three months at least, so that whatever Gout is in the system may be thrown off.

Some gentle aperient medicine must also be taken previous to the administration of the Humulus in chronic Gout, and when that has operated, two drachms of the tincture and twenty grains of the extract must be taken at two doses, viz. at night and at noon, (in pills, with draughts or mixtures) daily for the first month, three drachms and thirty grains for the second month, and two drachms and twenty grains for the third month, which quantity in general will be amply sufficient to strengthen the system, and expel all the present Gout. At any future time, when more Gout is generated, and accumulates sufficiently to produce a paroxysm, attended with fever, pain, and inflammation in the affected part, it must be treated as acute Gout, but it will seldom last many days if attended to directly.

Whatever sufferings patients may have endured, when restored to health, they are too apt to be negligent with respect to keeping the bowels clear from crudities, that may gradually collect; therefore, I strongly recommend persons who are subject to Gouty affections, never to fail, however healthy they may appear, taking an aperient draught or

 pills pills every month or six weeks, as from experience I am convinced, that a paroxysm of Gout has often been prevented by such attention.

The regimen to be observed by persons suffering from chronic Gout will of course vary, according to the symptoms and constitution, as well as habits of the patient, which being known, can best be regulated by the attending medical gentleman. All however should attentively observe moderation, and cautiously avoid excess of every kind.

Those patients who have suffered from chronic rheumatism, have generally been relieved by the plan above directed.

---

*Memoirs of the Life of* THOMAS BEDDOES, *M. D. with an analytical Account of his Writings.* By JOHN EDMONDS STOCK, M. D. &c. &c. 4to. pp. 413. Murray. 1811.

WITHOUT preface or comment, we shall present our Readers with the most interesting particulars of this valuable work; not doubting but they will be gratified with the memoirs of a man, whose serious hours were intensely devoted to the attainment and propagation of medical science; whilst his lighter moments were happily directed to the cultivation of polite literature.

Thomas Beddoes was born of respectable parents, at Shiffnall, on the 13th day of April, 1760. He received the first rudiments of his education at a school in his native town, and from thence was removed to a seminary at Brood, in Staffordshire. He read perfectly well when five years old. An insatiable thirst for books, and a disinclination to partake of the usual amusements of children of his own age, characterized his infant years.—At the age of nine, he was placed at the free grammar-school in Bridgnorth, where he remained nearly four years; and made considerable progress in classical learning. He did not participate in the amusements of his young associates; but in the season allotted to recreation, was accustomed to walk round the play-ground with an air of thought and reflection which frequently excited the attention of his playful companions, who "wondered why he was always thinking."

Upon leaving this seminary he was placed under the tuition of an eminent scholar, the Rev. Samuel Dickenson, Rector of Plymhill, in Staffordshire, to be prepared for the University. Mr. Dickenson remarked, that whilst under his care young Beddoes was so intent upon literary pursuits, and especially classical learning, that he never devoted a single hour to diversions or frivolous amusements of any kind, whilst his moral conduct was irreproachable.

In 1776, he entered at Pembroke College, Oxford; continued tinued his habits of unremitting application, and soon established his reputation as a classical scholar. He acquired a competent knowledge of the French, German, and Italian languages, without the aid of a master. Having received a bias at his early age for the medical profession, he began to cultivate chemistry with ardour. The discoveries made about that period, by Black, and by Priestley, powerfully impressed his mind. He studied the subject deeply, and soon became acquainted with all that was known of pneumatic chemistry. He then proceeded to the study of mineralogy and of botany, and in both made rapid advances.

"His vacations at this period were generally spent in Shropshire, and he devoted much of his time to shooting and whist. To both these amusements he was equally partial. In his long morning rambles with his gun, he was accustomed to unite scientific research with his amusements. He explored every recess of the most rugged mountains; he searched every dell, and seldom returned home without his pockets filled with mineralogical or botanical specimens. As a whist-player, he was in much request: he was supposed to play that game as well as almost any man in England. He sometimes on these occasions amused his friends by a surprising effort of memory. He would relate, at the termination of a game, the exact order in which all the cards were placed, and particularize who had played them." P. 10.

In the year 1781, having taken his degree of Bachelor of Arts, Mr. Beddoes repaired to London, and entered upon the study of anatomy under the celebrated Sheldon. Having diligently dissected and acquired a knowledge of physiology, he attended the classes of the most distinguished lecturers in the metropolis, in other branches of the profession. About this period he executed a spirited translation of the physiological dissertations of Spallanzani. In a second edition, he added several observations of his own, and experiments instituted by others to illustrate the subjects treated of in the work.

He took his Master of Arts degree in 1783, and in the autumn of the following year proceeded to Edinburgh, and continued, during three successive winters and one summer, to prosecute his studies in the celebrated medical school of that city.

He soon distinguished himself in the Royal Medical, and Natural History Societies; of both of which he was elected President in the ensuing session.

In 1786, he returned to Oxford, and was admitted Doctor of Medicine on the 13th of December. In the spring, however, he again visited Edinburgh, where he remained some months, and afterwards made an excursion to the Highlands, during which he amused himself in botanizing and in collecting mineralogical specimens. Whilst on this tour, he accidentally dentally witnessed the beneficial effects of cold immersion in fever. Before ascending the mountain Schehallien, he had prescribed for a young woman labouring under a low fever then prevalent in the country, and which had in her case manifested symptoms of peculiar violence.

"On his return he was surprised to find her fever subdued, and no traces of indisposition remaining except weakness. It appeared that in her delirium she had uttered a wish for cold water, which being refused her, she had crawled, during the absence of her attendant, to the brink of an adjoining river; from which she immediately perceived a herd of cattle, with the drovers at some distance, on their way to cross the bridge. The sight induced her to make for the water, in hopes of concealing her nakedness. She waded up to her middle, and leaned against a fragment of rock; nor was it till one of the drovers turned his horse towards the inn, that she was discovered in this position; and it was believed that she had occupied it not less than five minutes. Her delirium was gone, and the symptoms of fever had quitted her."—P. 16.

In the autumn of this year Dr. B. visited the Continent, and associated with Guyton de Morveau and Lavoisier. Shortly after his return, he obtained the chemical lectureship at Oxford.

His acquaintance with Dr. Darwin, about this period, is thus introduced by our biographer.

"A congenial spirit of philosophical inquiry, and a corresponding ardour for the improvement of medicine, procured for him the distinguishing notice and regard of this truly illustrious medical philosopher. A frequent and friendly epistolary intercourse was kept up between them, confined chiefly to medical and philosophical subjects. In the course of it, it appears that the proof sheets of Zoonomia were regularly sent to Dr. Beddoes, and his opinions and criticisms were freely invited by the author. They seem to have been communicated with the same candour with which they had been solicited. He was not afraid to use the language of commendation, for the intrinsic merit of the work repelled the suspicion of flattery, and he offered his objections with as little reserve, because truth was the object of both."—P. 19—20.

It will readily be supposed that when Dr. Beddoes undertook to read lectures on chemistry at Oxford, he was not an idle occupant of the chair. In a letter to a friend he expresses the gratification he felt at the success of his lectures. He observes, "that they were attended by a full and overflowing audience, and that "the interest for scientific researches was not confined to the younger members of the University only." One of his pupils and friends, who was long connected with Oxford, speaks of the effect of his lectures in the following terms:

"The time of Dr. Beddoes' residence in Oxford was a brilliant one in the annals of the University. Science was cultivated more than it has been since, and I believe that I may say the same of the period which which preceded. Dr. Thomson's lectures on anatomy and mineralogy, and Dr. Sibthorp's on botany, were delivering at the same period; and produced a taste for scientific researches which bordered on enthusiasm.

*(To be continued.)*

---

*Practical Observations on the Sclerocele and other morbid Derangements of the* TESTICLE; *also on the Cause and Cure of the acute, the spurious, and the chronic* HYDROCELE. *The whole illustrated by Cases. To which are added, four Cases of Operation for* ANEURISM, *Subclavian, femoral, popliteal, and femoral-popliteal; with practical Remarks and Plates.* By THOMAS RAMSDEN, Surgeon to the Royal Foundation of Christ's Hospital, to the Foundling, and Assistant-Surgeon to Bartholomew's Hospital. 8vo. Lond. 1810. pp. xxiv. 347. Plates 2.

IF the opinion that medical literature is occupied, principally, by men of little practical knowledge, is founded on fact, it is not a doubtful assertion, that even this circumstance is accompanied with peculiar advantages. Though discoveries may not be made by this class of writers, though they may not suggest new modes of treating diseases on the ground of experience, they develope, by their erudite labours, the pages of antiquity; they select, examine, and condense, the knowledge of past times; and they render accessible to those, whose days and nights are employed in hurried visits to the sick, facts and observations which men of extensive practice have not leisure to seek among the frightful multitude of learned, dull, ingenious, obscure, illiterate, or trifling books.

In the instance before us we have the satisfaction to notice, however, a book written by a Gentleman of extensive practice, of undoubted chirurgical skill, and of deserved reputation for operative dexterity.

As it is our intention to pass by, as much as we can, all hypotheses, and to dwell upon practical facts, and that intelligence which leads to new and improved methods of treating diseases, we shall touch but slightly on the Preface or introductory chapter of Mr. Ramsden's work, which contains what he denominates "a new theory with regard to schirrus and cancer of the Testicle." But it will be proper to observe, that we are instructed in this prefatory part, that accident directed its author to an inquiry which ended in a conclusion, that, *most of the cases of enlarged and indurated Testicle are symptomatic.*

"While it is not my intention," he says, "to deny the existence of idiopathic diseases in the testicle, I am fully authorized in asserting, that such a disease is extremely rare, and that very many cases of morbid induration, induration, hitherto supposed to be idiopathic, may be safely considered, and successfully treated, *as arising from a principle of irritation concealed within the urethra.*"

In pursuing this investigation, the object is to shew that—

"Irritation frequently applied to a testicle, will produce appearances and consequences very similar to what are esteemed true characteristics of schirrus and carcinoma. And it is deduced from this fact, that the *malignancy of the ulcerative stage of true schirrus in the testicle* does not, as has been supposed, depend on the presence of any morbid poison, but differs from the *malignancy of the ulcerative stage of common indurated testicle*, merely with regard to the part of the gland in which irritation causing its derangement has been primarily established.

"In illustrating this opinion, it is to be remarked that when a testicle is affected by true schirrus, as it is termed, its morbid alteration will be found to originate within its organic structure; but when the gland becomes indurated and enlarged in consequence of exterior causes of excitement, the morbid symptoms are, in the first instance, entirely confined to the surrounding or intervening cellular substance."

This is not to be considered as an hypothesis founded on conjecture, or arising out of analogical deductions, but as a plain demonstrable fact.

"If a testicle, enlarged and indurated by idiopathic, schirrous derangement, be divided and examined, its organic structure, even before the gland has become painful or inflamed, will be found imperfect or obscured; the centre is more compact and has a more uniform texture than the rest of the tumour, and is nearly the consistence of cartilage. This middle part does not exceed the size of a silver penny, and from this in every direction, like rays, are seen ligamentous bands of a white colour and very narrow, looking in the section like so many irregular lines passing to the circumference of the tumour, which is blended with the substance of the surrounding gland. In the interstices between these bands the substance is different, and becomes less compact towards the edges."

"If a testicle, indurated and enlarged from excitement exterior to itself, be examined before it becomes painful or inflamed, the morbid alteration will be found in the cellular substance only, and will appear more and more faint as it approaches near to the organic structure, which is yet entire."

Upon these premises, and admitting there are two very distinct classes of enlarged and indurated testes, symptomatic and idiopathic, Mr. Ramsden states, with a propriety of limitation that becomes the investigator of the laws of nature, and the inquirer after truth,

"That the disease which we are accustomed to call true schirrus in the testicle, consists solely in irritation primarily established within the organic structure,* and that such idiopathic induration differs from symptomatic induration in the following particulars: the former always

* By organic structure those parts of a gland are meant which are necessary to its particular functions, and the office which it holds in the animal economy.

proceeding proceeding FROM organic structure towards the surrounding cellular substance; the latter as uniformly proceeding from the surrounding and intervening cellular substance TOWARDS organic structure."

In conformity with this arrangement Mr. Ramsden proposes to distinguish these different forms of morbid alteration in the testicle, by the terms

SCLEROCELE, for the symptomatic induration.

IDIO-SCLEROCELE, for the idiopathic induration.

HYDRO-SCLEROCELE, when the symptomatic induration is accompanied with a serous effusion into the tunica vaginalis testis*.

CARCINOMA, if retained at all, may be used to describe the ulcerative stage of symptomatic induration; and

IDIO-CARCINOMA, to express the ulcerative stage of idiopathic ulceration.

There is another form of disease in the Testicle, known by the term *Sarcocele*, upon which Mr. Ramsden treats, though he has not comprehended it in this arrangement. This morbid enlargement of the testicle, which appears distinctly to differ from the preceding, he thus describes.

"The Sarcocele, which has been unfortunately (I say unfortunately, because I believe it to be a circumstance which has perplexed and confounded our enquiries) considered by our best authors merely as a variety of Schirrus, in fact possesses very few morbid characters in common with it; it is true, indeed, that the Sarcocele commences within the organic structure of the gland, and proceeds according to the laws of common irritation, but in outward character and on dissection it displays features which are peculiar to itself; since it is fleshy and elastic to the feel, and when divided is often found to contain within its substance partial collections of bloody sanies; it is also in a great measure freed from that ligamentous radiated appearance, which, in true schirrus, is uniformly observable."

Having premised this statement as explanatory of the fundamental principles in Mr. Ramsden's work, we shall proceed in our next Number to present our Readers with an analytical view of such diseases of the testicle as are detailed by the author, under the general head of affections of the testicle dependent on a principle of latent irritation within the urethra, particularly as arising from a *membranous fence* at the aperture of the urethra—of the sclerocele, or indurated testicle—of the schirrous testicle, and its morbid distinctions—of the morbid state of the testicle which has been called venereal—of the scrophulous testicle—of the sarcocele—of the acute, spurious, and chronic hydrocele.

*(To be continued.)*

---

* Does the lymphatic effusion ever accompany the Idio-Sclerocele? If it does it will require, consistent with this nomenclature, a term to express it.

